# Antibiotic use in patients with severe acute respiratory syndrome due to SARS-CoV-2 in a Brazilian University Hospital (2020–2021)

**DOI:** 10.1017/ash.2025.10289

**Published:** 2026-03-26

**Authors:** Valeria Paes Lima, Gustavo Adolfo Sierra Romero

**Affiliations:** 1 Center for Tropical Medicine; University of Brasilia, Brasília, Brazil; 2 Department of Infectious Diseases, University Hospital of Brasília (HUB), Brasília, Federal District, Brazil; 3 Institute for Health Assessment and Translation for Chronic and Neglected Diseases of High Relevance – IATS-CARE, Belo Horizonte, Minas Gerais, Brazil

## Abstract

**Background:**

The COVID-19 pandemic has profoundly affected healthcare systems worldwide. High and often inappropriate antimicrobial use has been reported in COVID-19 care, potentially increasing the risk of bacterial resistance and other adverse events. This study aimed to characterize and quantitatively assess antimicrobial use among Brazilian patients hospitalized with severe acute respiratory syndrome (SARS) due to SARS-CoV-2 infection.

**Methods:**

This retrospective observational cohort study included patients hospitalized with SARS caused by SARS-CoV-2 at the University Hospital of Brasília (HUB) during 2020 and 2021. Data on antimicrobial regimens, duration of therapy, and days of use were extracted from medical records.

**Results:**

The median age was 61 years (IQR, 49–72); most patients were unvaccinated against COVID-19 (76.3%), and comorbidities were highly prevalent (90.1%). Patients were stratified by clinical severity at hospital discharge: 301 (47.2%) were classified as Severe COVID-19 and 337 (52.8%) as Critical COVID-19. Greater clinical severity was consistently associated with increased antimicrobial exposure across multiple indicators, including the proportion of patients receiving antimicrobials, days of therapy (DOT), length of therapy (LOT), and the DOT/LOT ratio and an inverse association was observed for antimicrobial-free days (AFD). According to the World Health Organization (WHO) AWaRe classification, Watch-group antibiotics were most frequently prescribed (91.9% of patients); however, Reserve-group antibiotics showed the greatest increases in both frequency and duration of use with increasing disease severity.

**Conclusions:**

In this single-center Brazilian cohort, antimicrobial therapy was highly prevalent (94.4%). Higher clinical severity was strongly associated with greater antimicrobial exposure and fewer AFD.

## Introduction

COVID-19 was first described in December 2019 in Wuhan, China. The number of cases increased rapidly and progressively, leading the World Health Organization to declare a pandemic on March 11, 2020.^1^ Brazil ranks sixth worldwide in the cumulative number of COVID-19 cases (37.9 million) and second in total deaths (704,000) (World Health Organization, COVID-19 Dashboard).

COVID-19 is a viral disease; therefore, the routine use of antimicrobials is not recommended. Nevertheless, studies from around the world have reported a high prevalence of antimicrobial use among hospitalized patients with COVID-19, in contrast to the low prevalence of microbiologically confirmed infections.^2–13^

The clinical manifestations of SARS-CoV-2 infection itself—such as fever and respiratory symptoms—often prompt differential diagnoses with community-acquired pneumonia or even sepsis in severe cases. Furthermore, bacterial or fungal infections may occur as overlapping or complicating conditions during the viral illness, including co-infections present at admission, reactivation of latent infections, hospital-acquired infections, and opportunistic fungal infections.^14–16^

During the COVID-19 pandemic, increases in healthcare-associated infections and bacterial resistance have been reported, potentially related to disruptions in infection prevention practices in the context of high-intensity care, limited availability of intensive care-trained personnel, and shortages of personal protective equipment.^17–21^

Systematic monitoring and data collection on antimicrobial use are essential to improve the quality of prescribing in hospital settings. In a rapid review published in November 2020 about antimicrobial use in patients with COVID-19, the authors identified frequent prescription of antimicrobials and recommended considering the risks of antibiotic-related adverse effects, such as QT prolongation, which may be associated with the use of macrolides and fluoroquinolones. They also emphasized the importance of maintaining infection prevention measures in hospitals and recognize the benefits of antimicrobial stewardship programs.^22^

A more recent systematic review and meta-analysis published in 2022 found that most of the included studies (33 out of 43 publications—76.7%) were conducted in high-income countries, where antimicrobial consumption was significantly lower (58%, 95% CI 48–67%) compared with middle- and low-income countries (89%, 95% CI 82–94%).^23^

There are few Brazilian data in the literature providing a comprehensive analysis of antimicrobial use in patients with severe acute respiratory syndrome (SARS) caused by SARS-CoV-2, particularly those based on individual-level.^24^ A Brazilian multicenter cohort study of 2,054 patients hospitalized with COVID-19 in 2020 across 25 hospitals reported antibiotic use in 87.9% of cases.^12^ Similarly, a cohort from Manaus including 530 patients hospitalized through July 2021 found antimicrobial use in 86.8%.^13^ However, these studies did not assess the duration of antimicrobial therapy, AWaRe-stratified use by severity, or density metrics per 1,000 patient-days.

This study aimed to characterize and quantitatively assess antimicrobial use in a cohort of Brazilian patients hospitalized with SARS due to SARS-CoV-2 infection. The primary outcome was antibiotic days of therapy (DOT). Secondary outcomes included length of therapy (LOT), the DOT/LOT ratio, and antimicrobial-free days (AFD). Analyses were further stratified according to clinical severity and the World Health Organization (WHO) AWaRe classification of antimicrobials.

## Methods

### Study design

This was a historical cohort observational study including patients hospitalized with SARS caused by SARS-CoV-2 at the University Hospital of Brasília (HUB) during 2020 and 2021.

#### *Severe acute respiratory syndrome* (SARS)

An individual with influenza-like illness presenting with dyspnea or respiratory distress OR persistent chest pressure OR oxygen saturation ≤94% OR bluish discoloration of the lips or face.

#### *Influenza-like illness* (ILI)

An individual with fever (including self-reported) accompanied by cough or sore throat, with symptom onset within the previous seven days.

### Patient inclusion

Patients were identified through case notifications from the Epidemiological Surveillance Unit of the University Hospital of Brasília. Active case finding was also conducted among hospitalized patients.

#### Inclusion criteria

- Age ≥18 years AND.
- Presence of SARS with laboratory-confirmed SARS-CoV-2 infection, as determined by PCR or antigen testing, using a nasopharyngeal swab or lower respiratory tract sample AND.
- Hospitalization at the University Hospital of Brasília (HUB) during 2020 and 2021.

#### Exclusion criteria

- Symptoms not attributable to SARS-CoV-2 infection (e.g., COVID-19 prior to hospital admission).
- Inter-hospital transfer within 24 hours of admission.

### Data sources

Patient information was collected from the date of first hospital admission through the date of discharge from the University Hospital of Brasília. For the hospitalization period prior to admission to HUB, data were obtained from transfer reports. After admission, clinical data were extracted from medical progress notes in the electronic medical records, and information on antimicrobial use was obtained directly from electronic prescriptions.

#### Final classification of the COVID-19 episode

Classification was determined at hospital discharge. Patients were stratified into:

- Severe COVID-19: Patients without a supplemental oxygen requirement or requiring supplemental oxygen with or without noninvasive ventilation.
- Critical COVID-19: Patients requiring invasive mechanical ventilation or those who died during hospitalization.

#### Antimicrobial use

Patients received treatment for COVID-19 in accordance with World Health Organization (WHO) protocols. During the study period, no specific antiviral therapy for COVID-19 was available.

The indication for antimicrobial therapy was determined by the attending medical team based on clinical assessment and complementary investigations. Laboratory parameters, including complete blood count, C-reactive protein, procalcitonin, imaging results, and microbiological tests were available throughout the study period. The Infectious Diseases team provided therapeutic guidance through participation in multidisciplinary ward rounds held three times per week, as well as through the involvement of infectious disease specialists integrated into the attending medical staff.

- Systemic antibacterial and antifungal agents administered orally or intravenously were included.
- A day of therapy (DOT) was defined as any calendar day during which a patient received a specific antimicrobial, regardless of dose or frequency. The variable DOT represents the sum of therapy days for each individual antimicrobial agent. Each day of administration of fixed-dose combination agents was counted as one day of therapy (DOT) (e.g., piperacillin–tazobactam).
- LOT represented the total number of days during hospitalization in which the patient received any antimicrobial, irrespective of the number of different agents administered on the same day.
- DOT/LOT ratio was calculated by dividing the total number of *DOT* by the *LOT* for each patient.
- An antimicrobial-free day (AFD) was defined as any day during hospitalization when the patient did not receive any antimicrobial therapy. The variable AFD corresponds to the total number of hospitalization days without antimicrobial therapy.
- Antimicrobials were categorized according to the World Health Organization (WHO) AWaRe classification, using the 2023 update (Access, Watch, and Reserve groups).
- Antimicrobial use indicators per 1,000 patient-days were calculated for the overall cohort and stratified by clinical severity. The formula used was the sum of days for each respective indicator (DOT, LOT, AFD, days of use for each antibiotic group according to the WHO AWaRe classification), divided by the total number of patient-days in the corresponding category, and multiplied by 1,000.

Other definitions are available in the Supplementary Material.

### Data validation

Upon completion of data collection, the database underwent validation procedures that included checking for duplicate records, confirming adherence to inclusion criteria, validating hospitalization outcomes and final case severity classification, and performing data consistency analyses.

#### Statistical analysis

Data were analyzed using IBM SPSS Statistics software.

Descriptive statistics were generated for the entire cohort and stratified by clinical severity. For numerical variables, summary measures such as mean, standard deviation, median, and interquartile range were calculated. Normality was assessed using the Kolmogorov–Smirnov and Shapiro–Wilk tests. When data were not normally distributed, the nonparametric Mann–Whitney U test was applied; for normally distributed data, analysis of variance (ANOVA) was used. For categorical variables, absolute and relative frequencies were reported, and associations were evaluated using Pearson’s χ^2^ test. A *P* value <.05 was considered statistically significant.

### Ethical considerations

The study was reviewed and approved by the Research Ethics Committee of the Faculty of Medicine at the University of Brasília (CAAE 32,964,820.5..5558). It was conducted in accordance with Brazilian and international ethical regulations, and patient confidentiality was strictly maintained throughout the research process.

## Results

**Figure 1.**
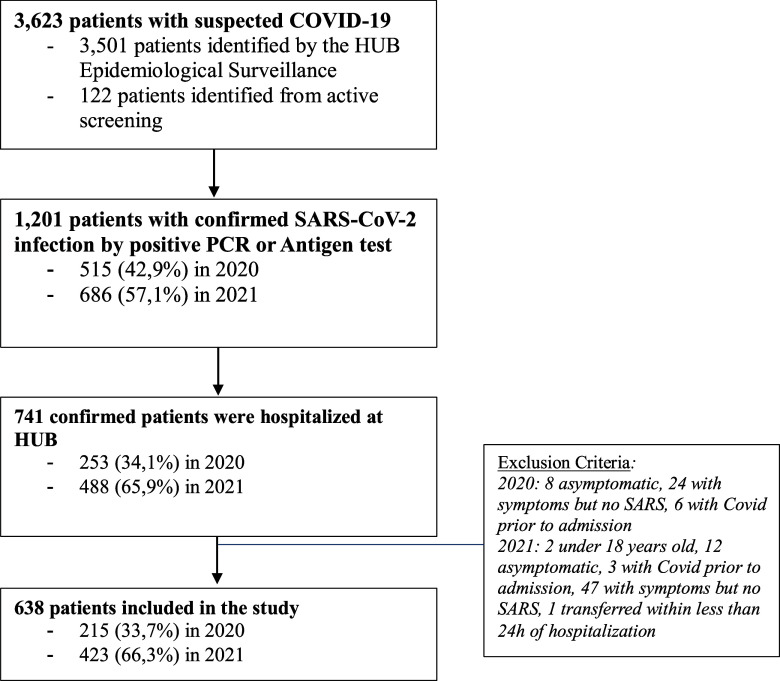
Study flowchart describing the recruitment pathway for participating patients.

During the study period, 3,623 patients with suspected COVID-19 were identified at the University Hospital of Brasília. A total of 638 patients met the inclusion criteria and were included in the final analysis, as shown in Figure 1. Eighty-one (12.7%) patients were admitted directly to the University Hospital of Brasília (HUB), whereas 557 (87.3%) were transferred from other healthcare institutions.

The general characteristics of the patients are presented in Table 1. The median age was 61 years (IQR 49–72), and 382 (59.9%) were male at birth. The majority identified as mixed race (350; 59.4%), 43 (6.7%) were current smokers, and 487 (76.3%) had not received prior COVID-19 vaccination. The median body mass index (BMI) was 26 (IQR 23–31). The infection was community-acquired in 263 (87.6%) cases. A total of 575 patients (90.1%) had at least one comorbidity and the median Charlson Comorbidity Index was 1 (IQR, 1–3). Information on the main comorbidities and vaccines received by the patients is available in the Supplementary Material.

**Table 1.** Baseline characteristics of patients hospitalized with severe acute respiratory syndrome due to SARS-CoV-2 at the University Hospital of Brasília (HUB), stratified by clinical severity, 2020–2021

	Overall cohort (*n* = 638)	Severe COVID-19 (*n* = 301)	Critical COVID (*n* = 337)	*P* value*
**Age (years)**	Median 61 (IQR 49–72)	Median 61 (IQR 50–71)	Median 61 (IQR 48–74)	.491
**Sex at birth**				.719
Female	256 (40.1%)	123 (40.9%)	133 (39.5%)	
Male	382 (59.9%)	178 (59.1%)	204 (60.5%)	
**Educational level**				.002
Incomplete primary education	103 (16.1%)	59 (19.6%)	44 (13.0%)	
Complete primary education	69 (10.8%)	27 (8.9%)	42 (12.5%)	
Complete secondary education	111 (17.4%)	57 (18.9%)	54 (16.0%)	
Complete higher education	50 (7.8%)	32 (10.6%)	18 (5.3%)	
Missing data	305 (47.8%)	126 (41.8%)	179 (53.1%)	
**Self-reported skin color**				.070
White	134 (21.0%)	61 (20.3%)	73 (21.7%)	
Black	47 (7.4%)	22 (7.3%)	25 (7.4%)	
Mixed race	350 (59.4%)	170 (56.5%)	180 (53.4%)	
Other	12 (1.9%)	10 (3.3%)	2 (.6%)	
Not recorded	95 (14.9%)	38 (12.6%)	57 (16.9%)	
**Smoking status**				.850
Current smoker	43 (6.7%)	22 (7.3%)	21 (6.2%)	
Former smoker	159 (24.9%)	76 (25.2%)	83 (24.6%)	
Never smoked	220 (34.5%)	99 (32.9%)	121 (35.9%)	
Missing data	216 (33.9%)	104 (34.6%)	112 (33.2%)	
**COVID-19 vaccination status**				.001
Unvaccinated	487 (76.3%)	210 (69.8%)	277 (82.2%)	
Incomplete vaccination	85 (13.3%)	51 (16.9%)	34 (10.1%)	
Complete vaccination	66 (10.4%)	40 (13.3%)	26 (7.7%)	
**Body mass index (BMI)**	Median 26 (IQR 23–31)	Median 27 (IQR 23–31)	Median 26 (IQR 23–30)	.219
**Probable site of infection**				.892
Community-acquired	559 (87.6%)	263 (87.4%)	296 (87.8%)	
Possible hospital-acquired	29 (4.5%)	13 (4.3%)	16 (4.7%)	
**Confirmed hospital-acquired**	50 (7.8%)	25 (8.3%)	25 (7.4%)	
Any comorbidity	575 (90.1%)	272 (90.4%)	303 (89.9%)	.848
Charlson Comorbidity Index	Median 1 (IQR 0–3)	Median 1 (IQR 0–2)	Median 1 (IQR 0–3)	.713

* significance *P* value for comparisons between severe and critical groups. χ^2^ test for proportions and Mann Whitney U-test for medians.

**Figure 2.**
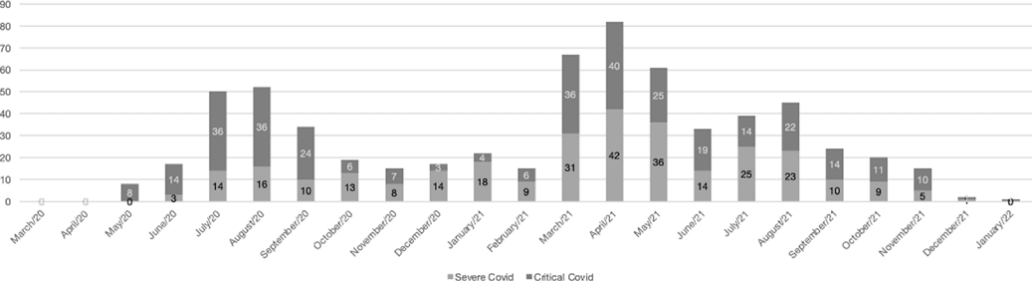
Number of patients hospitalized with severe acute respiratory syndrome (SARS) due to SARS-CoV-2 at Brasilia University Hospital (HUB) stratified by clinical severity and month of discharge, 2020–2021.

Patients were stratified according to clinical severity at the end of hospitalization, 301(47.2%) were classified as *Severe COVID-19*, and 337 (52.8%) as Critical COVID-19. Figure 2 shows the distribution of patients by clinical severity over time, according to the month of discharge.

When comparing the groups according to baseline characteristics, statistically significant differences were observed in educational level and vaccination status—critical patients were less likely to be vaccinated than severe patients. Due to the high proportion of missing data, conclusive comparisons of educational level between groups could not be performed.

Table 2 describes the clinical course of hospitalized patients. The median length of stay for the overall cohort was 20 days (IQR 12–36) and was significantly longer among patients in the Critical COVID-19 group (15 vs 24 d, *p* < .001). As expected, these patients also presented with a greater degree of pulmonary involvement on chest computed tomography and more frequent use of invasive interventions such as mechanical ventilation, hemodialysis, and vasopressor therapy. A total of 25 patients received palliative care, and all of them were in the Critical COVID-19 group. Most of those patients had advanced malignancies, dementia, or irreversible neurological conditions.

**Table 2.** Clinical outcomes of patients hospitalized with severe acute respiratory syndrome (SARS) due to SARS-CoV-2 at HUB, stratified by clinical severity, 2020–2021

Variable	Overall cohort (*n* = 638)	Severe COVID-19 (*n* = 301)	Critical COVID (*n* = 337)	p-Value
**Length of hospital stay (days)**	Median 20 (IQR 12–36)	Median 15 (IQR 10–26)	Median 24 (IQR 15–44)	<.001
**Maximum chest CT involvement**				<.001
0–25%	72 (11.3%)	59 (19.6%)	13 (3.9%)	
25–50%	144 (22.6%)	88 (29.2%)	56 (16.6%)	
50–75%	128 (20.1%)	51 (16.9%)	77 (22.8%)	
75–100%	77 (12.1%)	20 (6.6%)	57 (16.9%)	
Not quantified	174 (27.3%)	68 (22.6%)	106 (31.5%)	
No CT performed	43 (6.7%)	15 (5.0%)	28 (8.3%)	
**Mechanical ventilation**	305 (47.8%)	—	305 (90.5%)	—
**Vasopressor use**	242 (37.9%)	3 (1.0%)	239 (70.9%)	<.001
**Hemodialysis**	233 (36.5%)	51 (16.9%)	182 (54.0%)	<.001
**Palliative care**	25 (3,9%)	0	25 (7,4%)	<.001
**Hospital outcome**				<.001
Discharged home	388 (60.8%)	280 (93.0%)	108 (32.0%)	
Death	189 (29.6%)	0	189 (56.0%)	
Transferred to another hospital	61 (9.6%)	21 (7.0%)	40 (11.9%)	
**Antimicrobial use indicators**				
Days of Therapy (DOT)**	Median 17 (IQR 10–40)_	Median 10 (IQR 6–17)	Median 30 (IQR 16–62)	<.001
Length of Therapy (LOT)**	Median 11 (IQR 7–21)	Median 8 (IQR 5–12)	Median 17 (IQR 10–30)	<.001
DOT/LOT ratio**	Median 1.6 (IQR 1–2)	Median 1,2 (IQR 1–1.7)	Median 1.8 (IQR 1.4–2.3)	<.001
Antimicrobial-Free Days (AFD)**	Median 6 (IQR 1–14)	Median 7 (IQR 3–15)	Median 4 (IQR 0–14)	<.001

*significance *P* value for comparisons between severe and critical groups. χ^2^ test for proportions and Mann Whitney U-test for medians.

**Values were calculated among patients with complete antibiotic therapy information.

The case-fatality rate for the overall cohort was 29.6%, increasing to 56.0% among patients with Critical COVID-19.

### Antimicrobial use

A total of 602 patients (94.4%) received antimicrobials during hospitalization for SARS-CoV-2 infection—267 (88.7%) in the Severe COVID-19 group and 335 (99.4%) in the Critical COVID-19 group (*p* < .001).

Complete information on antimicrobial use—specifically, the number of days each antimicrobial agent was administered—was available for 620 patients (97.2%): 297 (98.7%) from the severe group and 323 (95.8%) from the critical group (*p* = .031). Subsequent analyses were performed for these patients.

**Figure 3.**
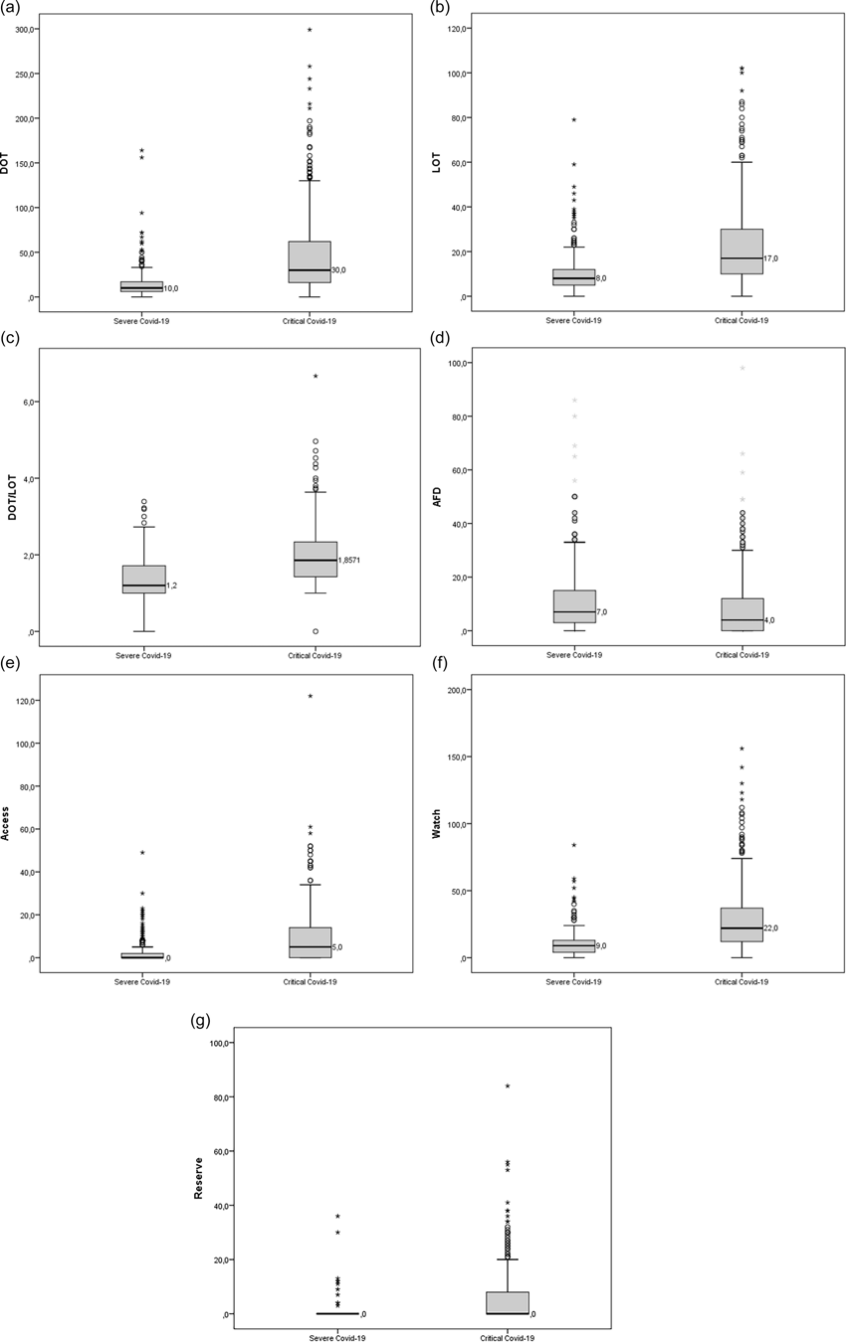
Boxplots of days (DOT) and duration (LOT) of antimicrobial use, DOT/LOT ratio, and antimicrobial-free days (AFD) (top), and boxplots of days of antimicrobial use according to the WHO AWaRe classification (bottom), among patients hospitalized with severe acute respiratory syndrome due to SARS-CoV-2 at the University Hospital of Brasília (HUB), stratified by clinical severity, 2020–2021.

Table 2 additionally presents median DOT, LOT, DOT/LOT ratio, and median AFD for patients in the overall cohort and in the Severe and Critical COVID-19 groups; these distributions are further illustrated in the boxplots presented in Figure 3. All indicators demonstrated higher mean levels of antimicrobial use among patients in the *Critical COVID-19* group, with statistically significant differences. The Supplementary Material reports the number of antibiotics used per patient in the severe and critical groups and includes adjusted analyses excluding palliative care patients.

An additional analysis was performed according to antimicrobial groups defined by the WHO AWaRe classification, as also illustrated in boxplots in Figure 3. Across all antimicrobial categories, patients in the *Critical COVID-19* group exhibited higher mean values of antimicrobial use, with differences reaching statistical significance.

Table 3 presents the number and proportion of patients who received antibiotics according to the WHO AWaRe classification, along with their corresponding mean duration of use. Patients in the critical group showed a significantly higher proportion and longer mean duration of use across all three WHO AWaRe antimicrobial categories.

**Table 3.** Proportion and duration of antimicrobial use among patients hospitalized with severe acute respiratory syndrome (SARS) due to SARS-CoV-2 at University Hospital of Brasilia (HUB), stratified by clinical severity, 2020–2021

	Proportion of Use				Mean time of use (days)			
	Overall cohort (n = 620)	Severe COVID-19 (n = 297)	Critical Covid (n = 323)	Statistics*	Overall cohort (n = 620)	Severe COVID-19 (n = 297)	Critical Covid (n = 323)	Statistics*
Antimicrobial	n (%)	n (%)	n (%)	p-value	Mean (SD)	Mean (SD)	Mean (SD)	p-value
**AWaRe group**								
Access	306 (49,3%)	87 (29,3%)	219 (67,.%)	<.001	6.46 (11.32)	2.47 (5,57)	10.13 (13.78)	**<.001**
Watch	570 (91,9%)	251 (84,5%)	319 (98.8%)	<.001	20.76 (21.91)	10.73 (10,31)	29.98 (25.44)	**<.001**
Reserve	149 (24,0%)	11 (3.7%)	138 (42.7%)	<.001	3.47 (8.76)	.475 (3,11)	6.23 (11.08)	**<.001**
**Meropenem**	295 (47,6%)	44 (14,8%)	251 (77.7%)	**<.001**	13.2 (10.80)	10.11 (7,13)	13.74 (11.25)	.066
**Ceftriaxone**	358 (57,7%)	171 (57.6%)	187 (57.9%)	.936	6..05 (3.23)	6.66 (3.30)	5.50 (3.07)	**.001**
**Vancomycin (systemic)**	182 (29,4%)	29 (9.8%)	153 (47.4%)	**<.001**	10.76 (9.25)	10.96 (8.16)	10.72 (9.47)	.639
**Piperacillin-tazobactam**	253 (40.8%)	70 (23.6%)	183 (56.7%)	**<.001**	7.39 (4.56)	7.65 (3.28)	7.28 (4.96)	.096
**Azithromycin**	291 (46.9%)	120 (40.4%)	171 (52.9%)	**.002**	5.01 (2.32)	4.75 (2.44)	5.19 (2.21)	**.023**

* significance *P* value for comparisons between severe and critical groups. χ^2^ test for proportions and Mann Whitney U-test for medians.

A significantly higher proportion of patients in the Critical COVID-19 group received the specific antibiotics included among the five most frequently prescribed agents, except for ceftriaxone, for which the proportion was high and similar between groups. Comparison of the mean duration of antimicrobial use revealed no statistically significant differences between groups, except for ceftriaxone and azithromycin. The complete table with results for all antimicrobials used is available in the Supplementary Material.

Antimicrobial use indicators per 1,000 patient-days were calculated for the overall cohort and stratified by clinical severity. For the overall cohort, the *DOT* was 1,225.3, the *LOT* was 638.5, the *AFD* was 372.5, and the *DOT/LOT ratio* was 1.92. When stratified by disease severity, patients in the *Severe COVID-19* group exhibited lower levels of antimicrobial exposure, with a DOT of 690.7 and a LOT of 478.7 per 1,000 patient-days, compared with patients in the *Critical COVID-19* group, who demonstrated markedly higher values (DOT 1539.9; LOT 732.5). The density of AFD was inversely associated with disease severity, decreasing from 538.1 in the *Severe* group to 275.1 in the *Critical* group.

With respect to the WHO AWaRe classification, the consumption of *Access-group* antibiotics was 240.3 per 1,000 patient-days in the overall cohort, lower among patients in the *Severe COVID-19* group (118.9) and higher among those in the *Critical COVID-19* group (311.2). *Watch-group* antibiotics accounted for most of the antimicrobial use (770.9 overall; 515.8 in Severe; 920.7 in Critical), whereas *Reserve-group* antibiotics were prescribed less frequently (129.1 overall; 22.8 in Severe; 191.6 in Critical).

## Discussion

This study characterizes the clinical features, hospitalization outcomes, and antimicrobial use patterns of a cohort comprising 638 patients hospitalized with *SARS* due to *SARS-CoV-2* at a federal university hospital in Brazil’s Federal District. The institution provides care exclusively through the public Brazilian Unified Health System. Most hospitalizations occurred in 2021, reflecting the national epidemiological context of the early pandemic phase, during which the P.1 (Gamma) variant was predominant in Brazil.^25^

Most patients (487; 76.3%) had not received vaccination against COVID-19. The Brazilian national vaccination campaign was initiated in January 2021, midway through the study period, with a progressive rollout prioritizing high-risk populations.^25^ Patients in the *Severe COVID-19* group had a significantly higher proportion of vaccinated individuals, although the overall frequency remained low (13.3% vs 7.7% in the *Critical COVID-19* group; *p* = .001).

This study found that 94.4% of patients received antimicrobial therapy, a result consistent with the findings reported by Khan S. and colleagues for lower-income countries.^23^

Several studies conducted worldwide have likewise reported a high proportion of antibiotic use,^2–11,26,27^ particularly among patients with greater disease severity or those who succumbed during hospitalization.^2,8,9,11,27,28^ Two additional studies conducted in Brazil reported comparable findings, with antibacterial use observed in 95.6% and 96.0% of patients who died from COVID-19.^12,13^

When evaluating exposure to individual antibiotics, patients in the *Critical COVID-19* group received nearly all agents more frequently than those in the *Severe COVID-19* group, except for ceftriaxone, which was prescribed to 57.6% and 57.9% of patients in the severe and critical groups, respectively. The mean duration of antibiotic use was generally comparable between groups, except for ceftriaxone and azithromycin, for which treatment duration was shorter among critical patients, possibly reflecting therapeutic escalation. The high proportion of ceftriaxone and azithromycin use may be attributed to concern for concomitant bacterial pulmonary infection, as this regimen is recommended for severe community-acquired pneumonia.

In the present study, substantial use of antimicrobials across all three categories of the WHO AWaRe classification was observed. The *Watch* category predominated, being prescribed to 91.9% of patients. *Reserve-group* antibiotics—agents regarded as the last line of therapeutic defense—were administered to 24.0% of patients in the overall cohort.

Studies stratifying antibiotic use by the WHO AWaRe classification have reported substantial variability across settings, with Access and Watch groups predominating overall, greater Watch and Reserve use among patients with more severe disease, and markedly different patterns in low- and middle-income settings such as Pakistan, where Watch-group antibiotics were used disproportionately more frequently.^5,9,26^

Therefore, it is noteworthy that the present cohort exhibited a high proportion of antibiotic use from the Reserve category.

Our analyses confirm that clinical severity is an important determinant of antimicrobial consumption indicators and should be considered when interpreting antimicrobial use metrics, particularly in settings where these indicators are routinely reported as aggregated measures at the ward or hospital level.

### Strengths and limitations

The data set demonstrated a high level of completeness. The use of an individual-level database enabled a refined assessment of determinants of antimicrobial exposure—particularly clinical severity—with additional stratification according to the WHO AWaRe classification. Furthermore, the study was conducted in a representative setting that remains underrepresented in the scientific literature: a Brazilian federal university hospital in a middle-income country providing care exclusively through the Unified Health System (SUS).

Nonetheless, certain limitations should be acknowledged. Antibiotics may have been prescribed for non-respiratory indications during the same admission, and neither prescribing appropriateness nor antibiotic-related adverse events were assessed. Microbiological data were not consistently available for the full cohort, limiting correlation between antimicrobial use and microbiological confirmation. A point-prevalence analysis conducted among patients hospitalized in 2020 identified bacteremia prevalences of 14.9% in the COVID-19 intensive care unit and 8.8% in the general ward (information available in the Supplementary Material). However, laboratory information system data for 2021 were unavailable, precluding complete recovery of microbiological information.

The assessment of the appropriateness of antibiotic use was beyond the scope of this retrospective study. Other studies based on aggregated databases, including Brazilian data, have demonstrated increased antimicrobial consumption during the pandemic and its association with rising bacterial resistance.^29,30^

Although not directly assessed, our data may help estimate the impact of the COVID-19 pandemic on hospital antibiotic use and offer indirect insight into the effect of vaccination through reduced COVID-19–related hospitalizations.

## Conclusion

Antimicrobial therapy was highly prevalent, prescribed to 94.4% of patients. Increasing clinical severity was consistently associated with greater antimicrobial consumption across multiple indicators. Within the WHO AWaRe classification, Watch-group antibiotics were most frequently used (91.9%), and patients with Critical COVID-19 showed substantial exposure to Reserve-category antibiotics.

## Supporting information

10.1017/ash.2025.10289.sm001 Lima and Romero supplementary material Lima and Romero supplementary material

## Supplementary material

The supplementary material for this article can be found at https://doi.org/10.1017/ash.2025.10289.
