# Supplementary material for: Antibiotic use in patients with severe acute respiratory syndrome due to SARS-CoV-2 in a Brazilian University Hospital (2020–2021)
Source: Antimicrob Steward Healthc Epidemiol. 2026 Mar 26;6(1):e65. doi: 10.1017/ash.2025.10289 (PMC13104578; doi:10.1017/ash.2025.10289)
Supplement: Lima and Romero supplementary material [file S2732494X25102891sup001.docx]

**Supplementary Material**

Definitions

***COVID-19 Vaccination Status*:**

- **Fully vaccinated:** Two doses of vaccine. with the second dose administered more than 14 days before symptom onset.
- **Partially vaccinated:** Received at least one vaccine dose but did not meet the criteria for full vaccination.
- **Unvaccinated:** Did not receive any vaccine dose.

The vaccines received by the patients included ChAdOx1 nCoV-19 (Oxford–AstraZeneca), CoronaVac (Sinovac), and BNT162b2 (Pfizer–BioNTech). No patient received the Ad26.COV2.S (Johnson & Johnson) vaccine.

***Source of infection:***

- **Community-acquired infection:** Symptom onset before hospital admission or within seven days after admission.
- **Possible hospital-acquired infection:** Symptom onset between seven and 14 days after admission.
- **Definite hospital-acquired infection:** Symptom onset more than 14 days after hospital admission.

**Reference: Agência Nacional de Vigilância Sanitária (ANVISA).** Technical Note GVIMS/GGTES/ANVISA No. 04/2020: Guidelines for prevention and control measures for COVID-19 in health services. Updated June 25. 2024. Brasília. Brazil: ANVISA; 2024. Available at: <https://www.gov.br/anvisa/pt-br/centraisdeconteudo/publicacoes/servicosdesaude/notas-tecnicas/notas-tecnicas-vigentes/NOTATCNICAGVIMS0420covid1925.06.2024.pdf>.

**Supplementary Table 1 – Number of vaccine doses and vaccination schedules administered to patients hospitalized with severe acute respiratory syndrome (SARS) due to SARS-CoV-2 at the University Hospital of Brasília (HUB) during 2020 and 2021**

| **Vaccination schedule (WHO nomenclature)** | **Number of patients** | **% among vaccinated patients (n = 151)** | **% among overall cohort patients (n = 638)** |
| --- | --- | --- | --- |
| 1 dose of **CoronaVac (Sinovac)** | 15 | 9.9% | 2.4% |
| 1 dose of **CoronaVac (Sinovac)** <15 days before symptom onset | 5 | 3.3% | 0.8% |
| 1 dose of **ChAdOx1 nCoV-19 (Oxford–AstraZeneca)** | 46 | 30.5% | 7.2% |
| 1 dose of **ChAdOx1 nCoV-19 (Oxford–AstraZeneca)** <15 days before symptom onset | 5 | 3.3% | 0.8% |
| 1 dose of **BNT162b2 (Pfizer–BioNTech)** | 5 | 3.3% | 0.8% |
| 1 dose of **BNT162b2 (Pfizer–BioNTech)** <15 days before symptom onset | 2 | 1.3% | 0.3% |
| 2 doses of **CoronaVac (Sinovac)** | 45 | 29.8% | 7.1% |
| 2 doses of **CoronaVac (Sinovac)** (second dose <15 days before symptom onset) | 2 | 1.3% | 0.3% |
| 2 doses of **ChAdOx1 nCoV-19 (Oxford–AstraZeneca)** | 21 | 13.9% | 3.3% |
| 2 doses of **ChAdOx1 nCoV-19 (Oxford–AstraZeneca)** (second dose <15 days before symptom onset) | 4 | 2.6% | 0.6% |
| 2 doses of **BNT162b2 (Pfizer–BioNTech)** (second dose <15 days before symptom onset) | 1 | 0.7% | 0.2% |

**Supplementary Table 2**. **Comorbidities among patients hospitalized with Severe Acute Respiratory Syndrome (SARS) due to SARS-CoV-2 at the University Hospital of Brasília (HUB). stratified by clinical severity. 2020–2021**

|  | **Overall Cohort**  **(n=638)** | **Severe COVID-19 (n=301)** | **Critical COVID (n=337)** | **p-value*** |
| --- | --- | --- | --- | --- |
| Any comorbidity | 575 (90.1%) | 272 (90.4%) | 303 (89.9%) | 0.848 |
| Charlson Comorbidity Index | Median 1 (IQR 0–3) | Median 1 (IQR 0–2) | Median 1 (IQR 0–3) | 0.713 |
| **Specific comorbidities** |  |  |  |  |
| Systemic arterial hypertension | 381 (59.7%) | 184 (61.1%) | 197 (58.5%) | 0.492 |
| Diabetes mellitus | 228 (35.7%) | 102 (33.9%) | 126 (37.4%) | 0.357 |
| Obesity | 180 (28.2%) | 91 (30.2%) | 89 (26.4%) | 0.225 |
| Chronic kidney disease | 152 (23.8%) | 77 (25.6%) | 75 (22.2%) | 0.323 |
| Chronic pulmonar disease | 84 (13.2%) | 44 (14.6%) | 40 (11.9%) | 0.305 |
| Heart failure | 61 (9.6%) | 32 (10.6%) | 29 (8.6%) | 0.385 |
| Cardiac arrhythmia | 46 (7.2%) | 23 (7.6%) | 23 (6.8%) | 0.691 |
| Cerebrovascular disease | 53 (8.3%) | 22 (7.3%) | 31 (9.2%) | 0.395 |
| Previous myocardial infarction | 44 (6.9%) | 27 (8.9%) | 17 (5.0%) | 0.051 |
| Alcohol use disorder | 42 (6.6%) | 22 (7.3%) | 20 (5.9%) | 0.478 |
| Localized solid tumor | 33 (5.2%) | 17 (5.6%) | 16 (4.7%) | 0.614 |
| Rheumatologic disease | 25 (3.9%) | 16 (5.3%) | 9 (2.7%) | 0.085 |
| Renal transplant | 22 (3.4%) | 9 (2.9%) | 13 (3.9%) | 0.554 |
| Metastatic solid tumor | 17 (2.7%) | 5 (1.7%) | 11 (3.2%) | 0.137 |
| Palliative care | 25 (3.9%) | 0 | 25 (7.4%) | <0.001 |

* significance p-value for comparisons between severe and critical groups. Chi-square test for proportions and Mann Whitney U-test for medians.

**Supplementary Table 3. Proportion and duration of antimicrobial use among patients hospitalized with Severe Acute Respiratory Syndrome (SARS) due to SARS-CoV-2 at HUB, stratified by clinical severity. 2020–2021**

|  | **Proportion of use** | | | | **Mean time of use (days)** | | | |
| --- | --- | --- | --- | --- | --- | --- | --- | --- |
|  | **Overall Cohort(n=620)** | **Severe COVID-19 (n=297)** | **Critical Covid (n=323)** | **Statistics** | **Overall Cohort(n=620)** | **Severe COVID-19 (n=297)** | **Critical Covid (n=323)** | **Statistics*** |
| **Antimicrobial** | n (%) | n (%) | n (%) | p-value | Mean (SD) | Mean (SD) | Mean (SD) | p-value |
| **Access** Group Antibiotic | 306 (49.3%) | 87 (29.3%) | 219 (67.8%) | **0.000** | 6.46 (11.32) | 2.47 (5.57) | 10.13 (13.78) | **0.000** |
| **Watch** Group Antibiotic | 570 (91.9%) | 251 (84.5%) | 319 (98.8%) | **0.000** | 20.76 (21.91) | 10.73 (10.31) | 29.98 (25.44) | **0.000** |
| **Reserve** Group Antibiotic | 149 (24.0%) | 11 (3.7%) | 138 (42.7%) | **0.000** | 3.47 (8.76) | 0.475 (3.11) | 6.23 (11.08) | **0.000** |
| **Meropenem** | 295 (47.6%) | 44 (14.8%) | 251 (77.7%) | **0.000** | 13.2 (10.80) | 10.11 (7.13) | 13.74 (11.25) | 0.066 |
| **Ceftriaxone** | 358 (57.7%) | 171 (57.6%) | 187 (57.9%) | 0.936 | 6.05 (3.23) | 6.66 (3.30) | 5.50 (3.07) | **0.001** |
| **Vancomycin (systemic)** | 182 (29.4%) | 29 (9.8%) | 153 (47.4%) | **0.000** | 10.76 (9.25) | 10.96 (8.16) | 10.72 (9.47) | 0.639 |
| **Piperacillin-tazobactam** | 253 (40.8%) | 70 (23.6%) | 183 (56.7%) | **0.000** | 7.39 (4.56) | 7.65 (3.28) | 7.28 (4.96) | 0.096 |
| **Azithromycin** | 291 (46.9%) | 120 (40.4%) | 171 (52.9%) | **0.002** | 5.01 (2.32) | 4.75 (2.44) | 5.19 (2.21) | **0.023** |
| **Amikacin** | 150 (24.2%) | 5 (1.7%) | 145 (44.9%) | **0.000** | 9.58 (8.08) | 7.8 (8.81) | 9.64 (8.08) | 0.367 |
| **Polymyxin B** | 130 (21.0%) | 4 (1.3%) | 126 (39.0%) | **0.000** | 10.48 (7.81) | 15.5 (14.05) | 10.32 (7.58) | 0.441 |
| **Ampicillin-sulbactam** | 128 (20.6%) | 44 (14.8%) | 84 (26.0%) | **0.001** | 6.27 (3.80) | 6.68 (4.05) | 6.05 (3.67) | 0.442 |
| **Gentamicin** | 72 (11.6%) | 7 (2.4%) | 65 (20.1%) | **0.000** | 9.08 (5.56) | 9.42 (3.82) | 9.04 (5.74) | 0.489 |
| **Ertapenem** | 50 (8.1%) | 1 (0.3%) | 49 (15.2%) | **0.000** | 11.14 (8.60) | 3 | 11.30 (8.61) | 0.2 |
| **Trimethoprim-sulfamethoxazole** | 41 (6.6%) | 9 (3.0%) | 32 (9.9%) | 0.001 | 13.17 (13.03) | 16 (10.12) | 12.37 (13.77) | 0.242 |
| **Micafungin** | 46 (7.4%) | 2 (0.7%) | 44 (13.6%) | **0.000** | 11.26 (12.01) | 11.5 (3.53) | 11.25 (12.28) | 0.558 |
| **Tigecycline** | 52 (8.4%) | 3 (1.0%) | 49 (15.2%) | **0.000** | 9.92 (6.67) | 13 (1.73) | 9.73 (6.82) | 0.155 |
| **Levofloxacin** | 63 (10.2%) | 19 (6.4%) | 44 (13.6%) | 0.003 | 7.68 (6.86) | 5.42 (3.45) | 8.65 (7.73) | 0.145 |
| **Fluconazol** | 39 (6.3%) | 6 (2.0%) | 33 (10.2%) | **0.000** | 7.17 (5.82) | 6.5 (4.88) | 7.30 (6.03) | 0.805 |
| **Ciprofloxacin** | 35 (5.6%) | 5 (1.7%) | 30 (9.3%) | **0.000** | 7.11 (4.67) | 6.8 (1.78) | 7.16 (5.01) | 0.766 |
| **Vancomycin (oral)** | 23 (3.7%) | 1 (0.3%) | 22 (6.8%) | **0.000** | 9.52 (5.40) | 2 | 9.86 (5.26) | N/A |
| **Cefepime** | 26 (4.2%) | 6 (2.0%) | 20 (6.2%) | 0.010 | 7.96 (10.04) | 6.5 (3.01) | 8.4 (11.37) | 0.573 |
| **Metronidazole (oral)** | 24 (3.9%) | 7 (2.4%) | 17 (5.3%) | 0.061 | 7.04 (3.56) | 7.71 (2.92) | 6.76 (3.84) | 0.455 |
| **Clindamycin** | 23 (3.7%) | 11 (3.7%) | 12 (3.7%) | 0.994 | 6.65 (4.91) | 5.27 (3.63) | 7.91 (5.71) | 0.211 |
| **Oxacillin** | 12 (1.9%) | 4 (1.3%) | 8 (2.5%) | 0.308 | 12.75 (9.92) | 9 (7.34) | 14.62 (10.94) | 0.57 |
| **Metronidazole (systemic)** | 17 (2.7%) | 5 (1.7%) | 12 (3.7%) | 0.122 | 8.7 (4.13) | 8.4 (2.5) | 8.83 (4.74) | 0.851 |
| **Daptomycin** | 13 (2.1%) | 2 (0.7%) | 11 (3.4%) | **0.018** | 11 (9.16) | 6.5 (3.53) | 11.81 (9.73) | 0.641 |
| **Linezolid** | 20 (3.2%) | 5 (1.7%) | 15 (4.6%) | **0.037** | 6.7 (3.77) | 5.4 (3.64) | 7.13 (3.83) | 0.388 |
| **Amphotericin B** | 12 (1.9%) | 1 (0.3%) | 11 (3.4%) | **0.006** | 7.58 (4.79) | 8 | 7.54 (5.02) | 0.667 |
| **Amoxicillin-clavulanate** | 22 (3.5%) | 16 (5.4%) | 6 (1.6%) | 0.018 | 3.45 (2.26) | 3.5 (2.39) | 3.33 (2.06) | 0.914 |
| **Voriconazole** | 1 (0.2%) | 1 (0.3%) | 0 | 0.297 | 47 | 47 | 0 | N/A |
| **Ampicilin** | 5 (0.8%) | 0 | 5 (1.5%) | **0.031** | 8.8 (4.86) | - | 8.8 (4.86) | N/A |
| **Imipenem** | 4 (0.6%) | 0 | 4 (1.2%) | 0.054 | 5.75 (6.60) | - | 5.75 (6.60) | N/A |

**Supplementary Table 4. Results of antimicrobial consumption indicators excluding palliative care patients**

| **Variable** | **Overall Cohort**  **n=596****  **Median (IQR)** | **Severe COVID-19**  **n=297****  **Median (IQR)** | **Critical COVID**  **n=299****  **Median (IQR)** | **p-value*** |
| --- | --- | --- | --- | --- |
| **Days of Therapy (DOT)** | 17 (10 – 40) | 10 (6 – 17) | 34 (18 – 65) | p<0.001 |
| **Lenght of Therapy (LOT)** | 11 (7 – 21) | 8 (5 – 12) | 18 (10 – 31) | p<0.001 |
| **DOT/LOT ratio** | 1.6 (1 – 2) | 1.2 (1 – 1.) | 1.9 (1.4 – 2.4) | p<0.001 |
| **Antimicrobial Free Days (AFD)** | 6 (1 – 14) | 7 (3 – 15) | 4 (0 – 12) | p<0.001 |

* Significance p-value for comparisons between severe and critical groups (Mann Whitney U-test).

**Values were calculated among patients with complete antibiotic therapy information.

**Supplementary Table 5. Number of antibiotics used per patient during hospitalization**

| Number of antibiotics used per patient during hospitalization | Overall cohort  n=638* | Severe COVID-19 n=301* | Critical COVID n=337* | p < 0.001 |
| --- | --- | --- | --- | --- |
| 1 | 97 (15.2%) | 80 (26.9%) | 17 (5.3%) |  |
| 2 | 134 (21.0%) | 102 (34.3%) | 32 (9.9%) |  |
| 3 | 93 (14.6%) | 43 (14.5%) | 50 (15.5%) |  |
| 4 | 65 (10.2%) | 24 (8.1%) | 41 (12.7%) |  |
| 5 or more | 195 (30.6%) | 14 (4.7%) | 181 (56.0%) |  |

* Patients with complete information on antimicrobial use

**Supplementary Table 6. Point-Prevalence Analysis of Antimicrobial Use in COVID-19 Units at the University Hospital of Brasília in 2020**

| Year: 2020 | COVID-19 Ward  (n=34) | COVID Intensive Care Unit  (n=47) |
| --- | --- | --- |
| Antimicrobial Use | 14 (41,1%) | 43 (91,4%) |
| Monotherapy | 6 (42,8%) | 9 (20,9%) |
| Healthcare-associated infection under treatment | 7 (20,5%) | 20 (46,5%) |
| **Documented infectious focus in the medical record** (among patients receiving antibiotics) | Lung (11 - 78%)  Sepsis without a defined focus (3 - 21,4%) | Lung (33 - 76,7%)  Sepsis without a defined focus (8 - 18,6%)  Urinary tract (1 – 2,3%)  Central venous catheter infection (1 – 2,3%) |
| Most frequently prescribed antibiotics | Azithromycin (27%) e Ceftriaxone (18%)  9 different antibiotics prescribed | Meropenem (22,5%) e Polymyxin B (12,6%)  15 different antibiotics prescribed |
| Blood culture obtained | 8 (23,5%) | 41 (87,2%) |
| Positive blood cultures (excluding contaminants) | 3 (8,8%) | 7 (14,9%) |

Point-prevalence methodology: a cross-sectional assessment of all patients hospitalized in the hospital or within a specific unit, focusing on antimicrobial use.

Braz J Infect Dis. 2022;26(Suppl 2):102431. doi:10.1016/j.bjid.2022.102431.
